# Supplementary material for: Individual retrotransposon integrants are differentially controlled by KZFP/KAP1-dependent histone methylation, DNA methylation and TET-mediated hydroxymethylation in naïve embryonic stem cells
Source: Epigenetics Chromatin. 2018 Feb 26;11:7. doi: 10.1186/s13072-018-0177-1 (PMC6389204; doi:10.1186/s13072-018-0177-1)
Supplement: Supplementary file 11 — Additional file 11. Pattern analysis. [file 13072_2018_177_MOESM11_ESM.zip › Patterns analysis/figs/p45.pdf]

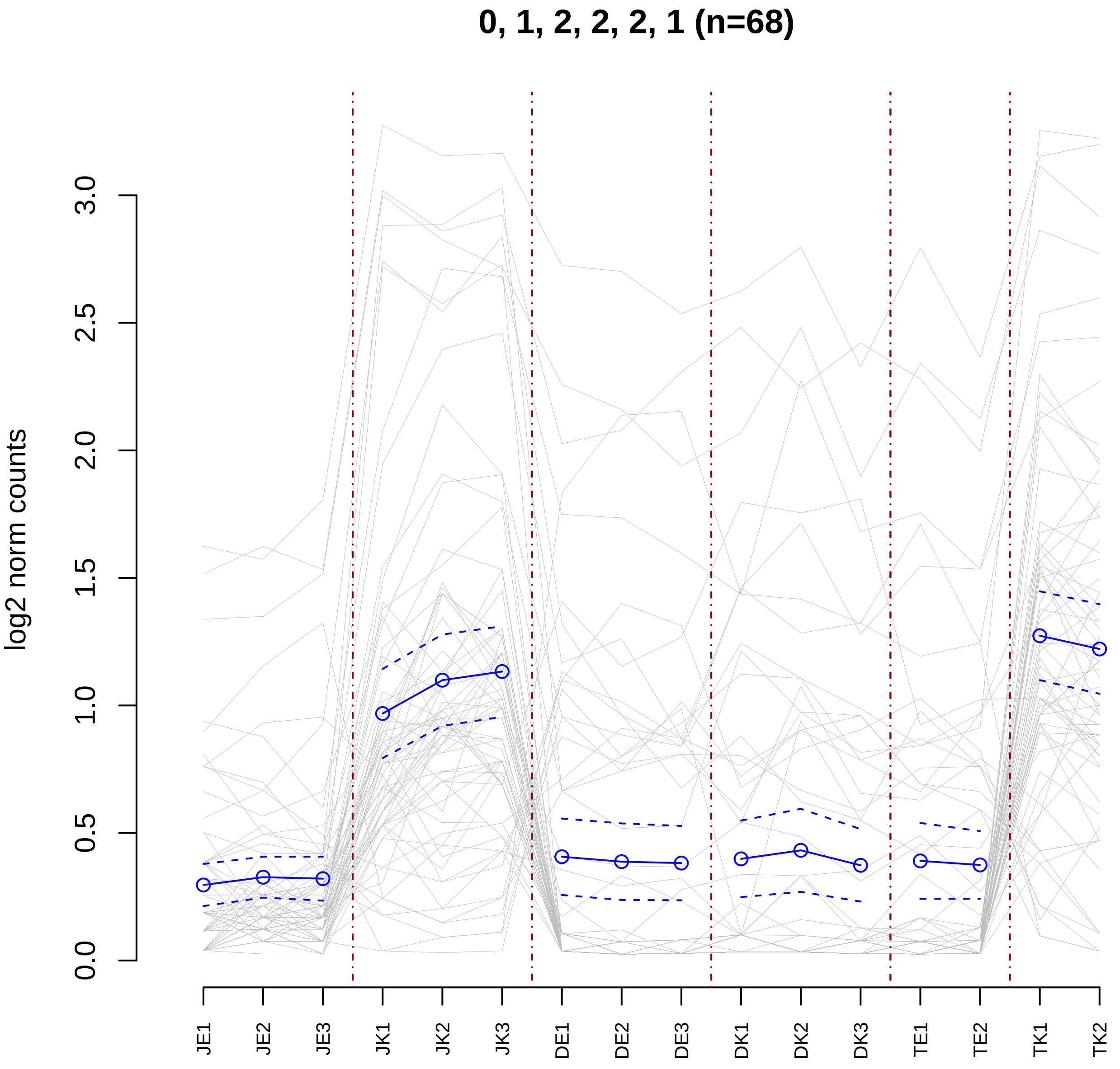

0, 1, 2, 2, 2, 1 (n=68)
log2 norm counts
0.0
0.5
1.0
1.5
2.0
2.5
3.0
JE1
JE2
JE3
JK1
JK2
JK3
DE1
DE2
DE3
DK1
DK2
DK3
TE1
TE2
TK1
TK2
